# 2-[2-(3-Methoxy­phen­yl)-2-oxoeth­yl]-1,2-benzisothia­zol-3(2*H*)-one 1,1-dioxide

**DOI:** 10.1107/S160053681000543X

**Published:** 2010-02-13

**Authors:** Salman Gul, Hamid Latif Siddiqui, Matloob Ahmad, Muhammad Azam, Masood Parvez

**Affiliations:** a Institute of Chemistry, University of the Punjab, Lahore, Pakistan; b Institute of Biochemistry, University of Baluchistan, Quetta 8700, Pakistan; c Department of Chemistry, University of Calgary, 2500 University Drive NW, Calgary, Alberta, Canada T2N 1N4

## Abstract

In the title compound, C_16_H_13_NO_5_S, the benzothia­zole unit is essentially planar [maximum deviation = 0.0501 (10) Å for the S atom] and is oriented at a dihedral angle of 67.85 (5)° with respect to the meth­oxy-substituted benzene ring. The mean plane of the meth­oxy group is oriented at 14.3 (3)° with respect to the benzene ring to which it is attached. In the crystal structure, weak C—H⋯O hydrogen bonds form macrocyclic rings with *R*_2_^2^(10) and *R*_2_^2^(12) motifs.

## Related literature

For the use of 1,2-benzisothia­zoline-3-one 1,1-dioxide (saccharine) as an inter­mediate in the preparation of medicinally important mol­ecules, see: Siddiqui *et al.* (2006 ▶); Zia-ur-Rehman *et al.* (2005 ▶, 2009 ▶). For the biological activity of saccharine, see: Singh *et al.* (2007 ▶); Vaccarino *et al.* (2007 ▶); Kapui *et al.* (2003 ▶). For related structures, see: Ahmad *et al.* (2008 ▶, 2009 ▶). For hydrogen-bonding motifs, see: Bernstein *et al.* (1995 ▶).
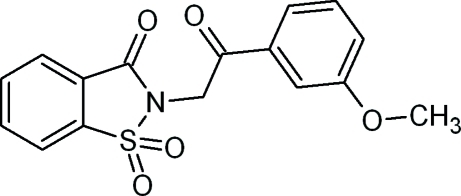


## Experimental

### 

#### Crystal data

C_16_H_13_NO_5_S
*M* *_r_* = 331.33
Monoclinic, 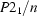

*a* = 8.9824 (3) Å
*b* = 8.5801 (4) Å
*c* = 19.5645 (7) Å
β = 97.942 (2)°
*V* = 1493.37 (10) Å^3^
*Z* = 4
Mo *K*α radiation
μ = 0.24 mm^−1^
*T* = 173 K
0.14 × 0.12 × 0.10 mm

#### Data collection

Nonius diffractometer with Bruker APEXII CCD
Absorption correction: multi-scan (*SORTAV*; Blessing, 1997 ▶) *T*_min_ = 0.967, *T*_max_ = 0.976
15084 measured reflections
3399 independent reflections
2897 reflections with *I* > 2σ(*I*)
*R*_int_ = 0.027

#### Refinement

*R*[*F* ^2^ > 2σ(*F* ^2^)] = 0.046
*wR*(*F* ^2^) = 0.136
*S* = 1.06
3399 reflections
209 parameters
H-atom parameters constrained
Δρ_max_ = 0.34 e Å^−3^
Δρ_min_ = −0.37 e Å^−3^

### 

Data collection: *COLLECT* (Nonius, 1998 ▶); cell refinement: *HKL* *DENZO* (Otwinowski & Minor, 1997 ▶); data reduction: *SCALEPACK* (Otwinowski & Minor, 1997 ▶); program(s) used to solve structure: *SHELXS97* (Sheldrick, 2008 ▶); program(s) used to refine structure: *SHELXL97* (Sheldrick, 2008 ▶); molecular graphics: *ORTEP-3 for Windows* (Farrugia, 1997 ▶); software used to prepare material for publication: *SHELXL97*.

## Supplementary Material

Crystal structure: contains datablocks global, I. DOI: 10.1107/S160053681000543X/lh2991sup1.cif

Structure factors: contains datablocks I. DOI: 10.1107/S160053681000543X/lh2991Isup2.hkl

Additional supplementary materials:  crystallographic information; 3D view; checkCIF report

## Figures and Tables

**Table 1:** Hydrogen-bond geometry (Å, °)

*D*—H⋯*A*	*D*—H	H⋯*A*	*D*⋯*A*	*D*—H⋯*A*
C5—H5⋯O5^i^	0.95	2.53	3.404 (3)	153
C8—H8*B*⋯O1^ii^	0.99	2.42	3.318 (3)	150
C8—H8*A*⋯O2^i^	0.99	2.51	3.301 (3)	137

Symmetry codes: (i) 
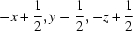
; (ii) 
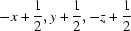
.

**Fig. 1.**
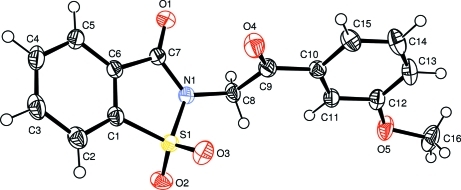
ORTEP-3 (Farrugia, 1997) drawing of (I) with displacement ellipsoids plotted at 50% probability level.

**Fig. 2.**
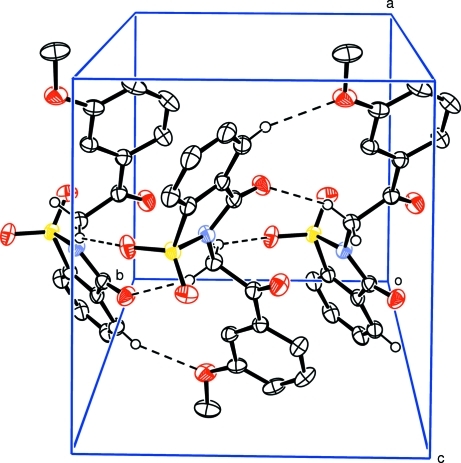
Unit cell packing of (I) showing non-classical hydrogen bonding interactions with dashed lines; H atoms not involved in H-bonds have been excluded for clarity.

## supplementary crystallographic information

### Comment

1,2-Benzisothiazoline-3-one 1,1-dioxide (saccharine) is an important starting material for the synthesis of different heterocyclic compounds and plays a role as an intermediate for the preparation of medicinally important molecules (Siddiqui *et al.*, 2006; Zia-ur-Rehman *et al.*, 2009). Various derivatives of saccharin are known to be cyclooxygenase-2 (COX-2) inhibitors (Singh *et al.*, 2007), analgesic (Vaccarino *et al.*, 2007), human leucocyte elastase (HLE) inhibitors (Kapui *et al.*, 2003) etc. In continuation of our research on the synthesis of potential biologically active derivatives of benzothiazines (Ahmad *et al.*, 2008; Ahmad *et al.*, 2009), we herein report the crystal structure of the title compound, *N*-(3-methoxyphenacyl)saccharin, (I).

The structure of (I) contains discrete molecules separated by normal van der Waals distances (Fig. 1). The benzothiazole moiety (S1/N1/C1–C7) is essentially planar (maximum deviation = 0.0501 (10) Å for atom S1) and lies at an angle 67.85 (5)° with respect to the benzene ring ). The methoxy group is oriented at 14.3 (3)° with respect to the benzene ring (C10–C15). The structure is devoid of any classical hydrogen bonds. However, non-classical hydrogen bonding interactions of the type C—H···O are present in the crystal structure resulting in ten and twelve membered macrocyclic rings in R_2_^2^(10) and R_2_^2^(12) motifs (Bernstein *et al.*, 1995) (Fig. 2 and Table 1).

### Experimental

3-Methoxy phenacyl bromide (5.49 g, 0.024 mol) was slowly added to a suspension of sodium saccharine (5 g, 0.024 mol) in dimethylformamide (15 ml) and the mixture was stirred at 383 K for 3.0 hours under anhydrous conditions. On completion of reaction (indicated by tlc), the mixture was poured on crushed ice and the precipitates formed were filtered and washed with an excess of distilled water and cold ethanol respectively. Crystals suitable for diffraction were grown from a solution of (I) in chloroform–methanol (3:1).

### Refinement

All H atoms were located from the difference Fourier maps and were included in the refinements at geometrically idealized positions with C—H distances = 0.95, 0.98 and 0.99 Å for aryl, methyl and methylene H atoms, respectively, and U_iso_ = 1.2 times U_eq_ of the C atoms to which they were bonded. The final difference map was free of chemically significant features.

### Crystal data

**Table:**

C_16_H_13_NO_5_S	*F*(000) = 688
*M**_r_* = 331.33	*D*_x_ = 1.474 Mg m^−^^3^
Monoclinic, *P*2_1_/*n*	Melting point: 446 K
Hall symbol: -P 2yn	Mo *K*α radiation, λ = 0.71073 Å
*a* = 8.9824 (3) Å	Cell parameters from 3447 reflections
*b* = 8.5801 (4) Å	θ = 1.0–27.5°
*c* = 19.5645 (7) Å	µ = 0.24 mm^−^^1^
β = 97.942 (2)°	*T* = 173 K
*V* = 1493.37 (10) Å^3^	Prism, white
*Z* = 4	0.14 × 0.12 × 0.10 mm

### Data collection

**Table:**

Nonius APEX2 CCD diffractometer	3399 independent reflections
Radiation source: fine-focus sealed tube	2897 reflections with *I* > 2σ(*I*)
graphite	*R*_int_ = 0.027
φ and ω scans	θ_max_ = 27.5°, θ_min_ = 2.6°
Absorption correction: multi-scan (*SORTAV*; Blessing, 1997)	*h* = −11→11
*T*_min_ = 0.967, *T*_max_ = 0.976	*k* = −11→11
15084 measured reflections	*l* = −25→25

### Refinement

**Table:**

Refinement on *F*^2^	Primary atom site location: structure-invariant direct methods
Least-squares matrix: full	Secondary atom site location: difference Fourier map
*R*[*F*^2^ > 2σ(*F*^2^)] = 0.046	Hydrogen site location: inferred from neighbouring sites
*wR*(*F*^2^) = 0.136	H-atom parameters constrained
*S* = 1.06	*w* = 1/[σ^2^(*F*_o_^2^) + (0.0682*P*)^2^ + 1.0411*P*] where *P* = (*F*_o_^2^ + 2*F*_c_^2^)/3
3399 reflections	(Δ/σ)_max_ = 0.001
209 parameters	Δρ_max_ = 0.34 e Å^−^^3^
0 restraints	Δρ_min_ = −0.37 e Å^−^^3^

### Special details

**Table:**

Geometry. All esds (except the esd in the dihedral angle between two l.s. planes) are estimated using the full covariance matrix. The cell esds are taken into account individually in the estimation of esds in distances, angles and torsion angles; correlations between esds in cell parameters are only used when they are defined by crystal symmetry. An approximate (isotropic) treatment of cell esds is used for estimating esds involving l.s. planes.

### Fractional atomic coordinates and isotropic or equivalent isotropic displacement parameters (Å^2^)

**Table:**

	*x*	*y*	*z*	*U*_iso_*/*U*_eq_	
S1	0.22107 (5)	0.29868 (6)	0.10828 (2)	0.02685 (16)	
O1	0.03462 (17)	−0.00650 (19)	0.20389 (8)	0.0382 (4)	
O2	0.21767 (18)	0.45904 (18)	0.12764 (8)	0.0368 (4)	
O3	0.35116 (16)	0.2458 (2)	0.08049 (8)	0.0391 (4)	
O4	0.36297 (18)	−0.07414 (19)	0.17637 (8)	0.0417 (4)	
O5	0.79500 (17)	0.2056 (2)	0.39967 (9)	0.0431 (4)	
N1	0.19250 (18)	0.1858 (2)	0.17480 (9)	0.0284 (4)	
C1	0.0523 (2)	0.2394 (2)	0.05901 (10)	0.0270 (4)	
C2	−0.0056 (3)	0.2914 (3)	−0.00668 (11)	0.0332 (5)	
H2	0.0427	0.3702	−0.0297	0.040*	
C3	−0.1387 (3)	0.2208 (3)	−0.03674 (12)	0.0389 (5)	
H3	−0.1832	0.2531	−0.0814	0.047*	
C4	−0.2074 (2)	0.1051 (3)	−0.00311 (12)	0.0398 (5)	
H4	−0.2966	0.0577	−0.0256	0.048*	
C5	−0.1486 (2)	0.0565 (3)	0.06313 (12)	0.0355 (5)	
H5	−0.1971	−0.0217	0.0865	0.043*	
C6	−0.0165 (2)	0.1264 (2)	0.09381 (10)	0.0283 (4)	
C7	0.0673 (2)	0.0893 (2)	0.16294 (10)	0.0285 (4)	
C8	0.3125 (2)	0.1633 (2)	0.23198 (10)	0.0283 (4)	
H8A	0.2684	0.1527	0.2754	0.034*	
H8B	0.3787	0.2561	0.2363	0.034*	
C9	0.4060 (2)	0.0187 (2)	0.22173 (10)	0.0288 (4)	
C10	0.5481 (2)	−0.0057 (2)	0.27013 (10)	0.0264 (4)	
C11	0.6073 (2)	0.1091 (2)	0.31602 (10)	0.0282 (4)	
H11	0.5553	0.2048	0.3187	0.034*	
C12	0.7438 (2)	0.0836 (3)	0.35828 (10)	0.0313 (4)	
C13	0.8174 (2)	−0.0582 (3)	0.35630 (11)	0.0403 (6)	
H13	0.9093	−0.0765	0.3856	0.048*	
C14	0.7554 (3)	−0.1730 (3)	0.31103 (12)	0.0422 (6)	
H14	0.8052	−0.2705	0.3101	0.051*	
C15	0.6230 (3)	−0.1486 (3)	0.26732 (11)	0.0353 (5)	
H15	0.5833	−0.2273	0.2359	0.042*	
C16	0.9474 (3)	0.2006 (4)	0.43204 (13)	0.0506 (7)	
H16A	0.9728	0.2990	0.4563	0.061*	
H16B	1.0139	0.1849	0.3969	0.061*	
H16C	0.9601	0.1143	0.4652	0.061*	

### Atomic displacement parameters (Å^2^)

**Table:**

	*U*^11^	*U*^22^	*U*^33^	*U*^12^	*U*^13^	*U*^23^
S1	0.0282 (3)	0.0253 (3)	0.0253 (2)	−0.00364 (18)	−0.00269 (18)	0.00023 (18)
O1	0.0353 (8)	0.0327 (8)	0.0443 (8)	−0.0046 (6)	−0.0030 (7)	0.0125 (7)
O2	0.0499 (9)	0.0246 (8)	0.0336 (7)	−0.0070 (7)	−0.0030 (7)	−0.0014 (6)
O3	0.0301 (7)	0.0489 (10)	0.0378 (8)	−0.0030 (7)	0.0034 (6)	−0.0015 (7)
O4	0.0422 (9)	0.0365 (9)	0.0421 (9)	0.0039 (7)	−0.0096 (7)	−0.0122 (7)
O5	0.0274 (8)	0.0521 (11)	0.0451 (9)	0.0011 (7)	−0.0117 (7)	−0.0092 (8)
N1	0.0269 (8)	0.0274 (9)	0.0282 (8)	−0.0031 (7)	−0.0056 (7)	0.0039 (7)
C1	0.0284 (9)	0.0232 (9)	0.0270 (9)	0.0035 (8)	−0.0046 (7)	−0.0029 (7)
C2	0.0395 (11)	0.0298 (11)	0.0278 (10)	0.0037 (9)	−0.0045 (8)	−0.0011 (8)
C3	0.0428 (12)	0.0342 (12)	0.0340 (11)	0.0091 (10)	−0.0145 (9)	−0.0054 (9)
C4	0.0314 (10)	0.0313 (12)	0.0505 (13)	0.0060 (9)	−0.0164 (9)	−0.0091 (10)
C5	0.0273 (10)	0.0269 (11)	0.0483 (12)	0.0001 (8)	−0.0080 (9)	−0.0007 (9)
C6	0.0266 (9)	0.0214 (10)	0.0343 (10)	0.0016 (7)	−0.0055 (8)	0.0007 (8)
C7	0.0259 (9)	0.0228 (9)	0.0346 (10)	0.0002 (7)	−0.0031 (8)	0.0020 (8)
C8	0.0276 (9)	0.0289 (10)	0.0253 (9)	0.0032 (8)	−0.0068 (7)	−0.0002 (8)
C9	0.0296 (10)	0.0276 (10)	0.0278 (9)	−0.0004 (8)	−0.0009 (8)	−0.0008 (8)
C10	0.0266 (9)	0.0270 (10)	0.0254 (9)	0.0008 (7)	0.0031 (7)	0.0019 (7)
C11	0.0238 (9)	0.0302 (11)	0.0299 (9)	0.0026 (8)	0.0007 (7)	0.0010 (8)
C12	0.0236 (9)	0.0417 (12)	0.0280 (9)	0.0020 (8)	0.0011 (8)	0.0021 (9)
C13	0.0283 (10)	0.0580 (15)	0.0335 (11)	0.0155 (10)	0.0005 (9)	0.0051 (10)
C14	0.0399 (12)	0.0450 (14)	0.0416 (12)	0.0209 (11)	0.0051 (10)	0.0012 (10)
C15	0.0396 (11)	0.0324 (11)	0.0341 (10)	0.0086 (9)	0.0053 (9)	−0.0009 (9)
C16	0.0268 (11)	0.080 (2)	0.0413 (12)	−0.0054 (11)	−0.0093 (9)	0.0013 (13)

### Geometric parameters (Å, °)

**Table:**

S1—O2	1.4286 (16)	C5—H5	0.9500
S1—O3	1.4291 (16)	C6—C7	1.489 (3)
S1—N1	1.6701 (17)	C8—C9	1.527 (3)
S1—C1	1.7555 (19)	C8—H8A	0.9900
O1—C7	1.212 (2)	C8—H8B	0.9900
O4—C9	1.215 (2)	C9—C10	1.496 (3)
O5—C12	1.364 (3)	C10—C11	1.389 (3)
O5—C16	1.427 (3)	C10—C15	1.403 (3)
N1—C7	1.390 (3)	C11—C12	1.398 (3)
N1—C8	1.455 (2)	C11—H11	0.9500
C1—C6	1.379 (3)	C12—C13	1.388 (3)
C1—C2	1.391 (3)	C13—C14	1.388 (4)
C2—C3	1.396 (3)	C13—H13	0.9500
C2—H2	0.9500	C14—C15	1.382 (3)
C3—C4	1.383 (4)	C14—H14	0.9500
C3—H3	0.9500	C15—H15	0.9500
C4—C5	1.394 (3)	C16—H16A	0.9800
C4—H4	0.9500	C16—H16B	0.9800
C5—C6	1.390 (3)	C16—H16C	0.9800
			
O2—S1—O3	117.07 (10)	N1—C8—H8A	109.3
O2—S1—N1	109.91 (9)	C9—C8—H8A	109.3
O3—S1—N1	109.52 (9)	N1—C8—H8B	109.3
O2—S1—C1	112.10 (9)	C9—C8—H8B	109.3
O3—S1—C1	112.87 (9)	H8A—C8—H8B	107.9
N1—S1—C1	92.63 (9)	O4—C9—C10	121.86 (19)
C12—O5—C16	117.68 (19)	O4—C9—C8	120.25 (17)
C7—N1—C8	123.05 (17)	C10—C9—C8	117.87 (16)
C7—N1—S1	115.04 (13)	C11—C10—C15	120.17 (19)
C8—N1—S1	119.92 (14)	C11—C10—C9	121.80 (18)
C6—C1—C2	123.07 (19)	C15—C10—C9	118.02 (18)
C6—C1—S1	110.15 (14)	C10—C11—C12	119.80 (19)
C2—C1—S1	126.75 (17)	C10—C11—H11	120.1
C1—C2—C3	116.1 (2)	C12—C11—H11	120.1
C1—C2—H2	122.0	O5—C12—C13	124.50 (19)
C3—C2—H2	122.0	O5—C12—C11	115.27 (19)
C4—C3—C2	121.6 (2)	C13—C12—C11	120.2 (2)
C4—C3—H3	119.2	C12—C13—C14	119.2 (2)
C2—C3—H3	119.2	C12—C13—H13	120.4
C3—C4—C5	121.4 (2)	C14—C13—H13	120.4
C3—C4—H4	119.3	C15—C14—C13	121.5 (2)
C5—C4—H4	119.3	C15—C14—H14	119.2
C6—C5—C4	117.6 (2)	C13—C14—H14	119.2
C6—C5—H5	121.2	C14—C15—C10	119.0 (2)
C4—C5—H5	121.2	C14—C15—H15	120.5
C1—C6—C5	120.31 (19)	C10—C15—H15	120.5
C1—C6—C7	113.22 (17)	O5—C16—H16A	109.5
C5—C6—C7	126.45 (19)	O5—C16—H16B	109.5
O1—C7—N1	123.93 (18)	H16A—C16—H16B	109.5
O1—C7—C6	127.41 (18)	O5—C16—H16C	109.5
N1—C7—C6	108.65 (17)	H16A—C16—H16C	109.5
N1—C8—C9	111.66 (16)	H16B—C16—H16C	109.5
			
O2—S1—N1—C7	120.14 (16)	S1—N1—C7—C6	−5.4 (2)
O3—S1—N1—C7	−109.92 (16)	C1—C6—C7—O1	−178.6 (2)
C1—S1—N1—C7	5.48 (16)	C5—C6—C7—O1	−0.4 (4)
O2—S1—N1—C8	−75.42 (17)	C1—C6—C7—N1	2.3 (2)
O3—S1—N1—C8	54.52 (17)	C5—C6—C7—N1	−179.5 (2)
C1—S1—N1—C8	169.92 (16)	C7—N1—C8—C9	70.4 (2)
O2—S1—C1—C6	−116.57 (15)	S1—N1—C8—C9	−92.72 (19)
O3—S1—C1—C6	108.65 (16)	N1—C8—C9—O4	−11.3 (3)
N1—S1—C1—C6	−3.83 (16)	N1—C8—C9—C10	170.25 (17)
O2—S1—C1—C2	65.5 (2)	O4—C9—C10—C11	171.5 (2)
O3—S1—C1—C2	−69.3 (2)	C8—C9—C10—C11	−10.0 (3)
N1—S1—C1—C2	178.3 (2)	O4—C9—C10—C15	−7.4 (3)
C6—C1—C2—C3	−0.7 (3)	C8—C9—C10—C15	171.06 (19)
S1—C1—C2—C3	176.93 (17)	C15—C10—C11—C12	1.6 (3)
C1—C2—C3—C4	−0.6 (3)	C9—C10—C11—C12	−177.32 (18)
C2—C3—C4—C5	1.6 (4)	C16—O5—C12—C13	13.8 (3)
C3—C4—C5—C6	−1.2 (3)	C16—O5—C12—C11	−166.2 (2)
C2—C1—C6—C5	1.1 (3)	C10—C11—C12—O5	177.70 (18)
S1—C1—C6—C5	−176.85 (17)	C10—C11—C12—C13	−2.3 (3)
C2—C1—C6—C7	179.45 (19)	O5—C12—C13—C14	−178.9 (2)
S1—C1—C6—C7	1.4 (2)	C11—C12—C13—C14	1.2 (3)
C4—C5—C6—C1	−0.2 (3)	C12—C13—C14—C15	0.8 (4)
C4—C5—C6—C7	−178.2 (2)	C13—C14—C15—C10	−1.5 (4)
C8—N1—C7—O1	11.6 (3)	C11—C10—C15—C14	0.3 (3)
S1—N1—C7—O1	175.52 (17)	C9—C10—C15—C14	179.3 (2)
C8—N1—C7—C6	−169.29 (17)		

### Hydrogen-bond geometry (Å, °)

**Table:**

*D*—H···*A*	*D*—H	H···*A*	*D*···*A*	*D*—H···*A*
C5—H5···O5^i^	0.95	2.53	3.404 (3)	153
C8—H8B···O1^ii^	0.99	2.42	3.318 (3)	150
C8—H8A···O2^i^	0.99	2.51	3.301 (3)	137

Symmetry codes: (i) −*x*+1/2, *y*−1/2, −*z*+1/2; (ii) −*x*+1/2, *y*+1/2, −*z*+1/2.
